# Occurrence of *Clinostomum* Metacercariae in *Oreochromis mossambicus* from Mashoko Dam, Masvingo Province, Zimbabwe

**DOI:** 10.1155/2018/9565049

**Published:** 2018-11-15

**Authors:** Casper Mutengu, Wilson Mhlanga

**Affiliations:** ^1^Department of Biological Science, Bindura University of Science Education, Bindura, Zimbabwe; ^2^Department of Natural Resources, Bindura University of Science Education, Bindura, Zimbabwe

## Abstract

Mashoko Dam is in Ago-ecological Region 4 in Zimbabwe. Five sampling sites were randomly selected and each site was sampled twice per month, for six months. A total of 180 *Oreochromis mossambicus* fish (101 females and 79 males) were caught. The fish were examined for *Clinostomum* metacercariae by cutting the ventral side from the anal opening to the lower jaw. The gill chambers were examined and inspected visually to detect macroscopic parasites. Of the 180 fish collected during the study, 113 (62.8%) were infected by 284 *Clinostomum* larvae in the cranial cavity while 67 fish were not infected. Among the infected fish, 46 were males and 67 were females. Greater parasite burden and mean intensity were observed in female fish (2.7 MI) than males (2.2 MI). There was no statistically significant difference in mean intensity of infection between male and female fish (*n*=180; *t*=0.521; *p* < 0.05). Uninfected fish were in a poorer condition than infected fish in July and October only. The lowest monthly condition factor for both infected (1.8) and uninfected (1.7) fish occurred in October. The monthly condition factors for both infected (1.94–3.51) and noninfected fish (1.81–5.28) were greater than 1. For prevalence by total length groups, highest prevalence (66.3%) was recorded in the medium length group (10–12 cm) and lowest (25.0%) in the (16–18 cm) length group. Highest mean intensity (2.8) and parasite density (146 parasites) was observed in the length group (13–15 cm) and lowest mean intensity (1.0) in larger length groups (16–18 cm and above 19 cm). Highest abundance (1.74) was recorded in the length group 13–15 cm and lowest abundance (0.25) in the length group 16–18 cm. Parasite burden was positively correlated to fish size (total length). It was concluded that *Clinostomum* metacercariae are a common parasite in *Oreochromis mossambicus* in Mashoko dam.

## 1. Introduction

Parasites are common among fishes, affecting them in a variety of ways [1]. Fish parasites are among the key threats to the sustainability of fisheries that support about 90 million people around the world as a source of protein and income [2]. Parasitic infections of fish have human health, as well as socioeconomic implications, both in developing and developed countries [3]. Parasites compete for food, thereby depriving fish of essential nutrients and inhibiting growth leading to morbidity and mortality with consequent economic losses [4]. Parasites also inflict damage on the hosts, sometimes causing gross mortalities, which can lead to great losses in commercial fisheries and aquaculture [5]. Fish parasites have the potential to affect fish through loss of blood and nutrients as well as the invasion of biochemical processes in the host fish [6]. In some cases, parasitised fish are so unsightly that they are rejected by consumers [6]. Studying parasites contributes towards a better understanding of the ecology of a system and also helps to develop appropriate methods of controlling them [7].

Parasites render fish susceptible to secondary infection by disease-causing agents such as bacteria, fungi, and viruses [8]. The extent of fish infection by certain parasites can be used as an early warning indicator of deteriorating water quality [9]. The use of indicators allows evaluating the risk of exposure, acting as early warning systems for environmental deterioration [10].

The life cycle of digeneans such as *Clinostomum* includes a molluscan host, fish (second intermediate host), and a definitive host which is usually a piscivorous bird [11]. Many species of freshwater fishes were recorded as the second intermediate host of *Clinostomum* metacercariae [12]. Juvenile fish, shallow water inhabitants, and bottom dwellers are the most vulnerable [13]. Most freshwater and estuarine fish are potential hosts for *Clinostomum* sp. metacercariae, and some warm-water fish species such as bluegill, largemouth, and catfish are also affected [13]. Aquatic birds also help in the dispersal of *Clinostomum* larvae [14]. Piscivorous birds such as herons, egrets, pelicans, and cormorants are the definitive hosts for many of the *Clinostomum* metacercariae found in fish [15]. Paperna [6] also reported that piscivorous birds that included the darter (*Anhinga rufa*) were definitive hosts for species of *Clinostomum.*

The description of *Clinostomum tilapiae* was done by Ukoli [16]. Ukoli [17] conducted life history and growth studies of *Clinostomum tilapiae*.

Heavy gill infection appears to lower respiratory efficiency [6]. While clinical effects of infection are usually not obvious, sudden, massive outbreaks of infection can be fatal [6]. In a study of parasites in the cichlid *Oreochromis mossambicus*, Olivier et al. [18] noted that, although clinostomid metacercariae cysts did not cause any deleterious effects in adult fish, severe infestation of *E. heterostomum* in juveniles resulted in locomotory impairment.

Echi and Ojebe [19] reported that there was a paucity of information regarding host specificity of clinostomatid infection. Generally, *Clinostomum* species do not display any host specificity, they seem to be more attracted to cichlids as compared to other species [19]. Immune resistance to clinostomatid infections is low, hence making them more susceptible to clinostomatid infections [19].

During the importation of fish, the movement and disposal of contaminated water, containers, and other equipment into rivers or dams contribute to the introduction and transport of parasites [20].

*Clinostomum* metacercariae can be observed as yellow cysts embedded in muscles or beneath the skin, especially at the base of the tail of fins in fish but heavy infestations are found in the body cavity, head, throat, and gills [21]. In a study of parasites in the cichlid *Tilapia zilli*, Echi et al. [22] noted that the *Clinostomum* microhabitats in *T. zilli* were the buccal cavity, eye, and skin, with the highest infection occurring in the buccal cavity. *Clinostomum* are also common in the caudal, dorsal, and pectoral fins, on the inside surface of the operculum and in the flesh of many fishes [21]. Digenetic trematodes are of great interest in many countries, especially for human health care against transmissible diseases [23].

Heavy infestations of *Clinostomum* (“yellow grub”) accumulate in freshwater fish which are classified by fishermen as unfit for human consumption [5]. The parasites are considered one of the most common parasites infecting fish, causing low weight, reduced marketability, and high mortality [5]. Yellow grub may kill fish under some circumstances, but normally fish are not noticeably affected by the parasite [13]. *Clinostomum* metacercariae species have zoonotic importance in the transmission of yellow grub disease to humans [6]. Humans get infected by the disease as a result of ingesting raw or improperly cooked fish [6]. The fluke becomes accidentally attached onto the surface of the mucus membranes of the throat and causes a clinical syndrome called halzoun [24]. Cooking of fish destroys the grub without altering the flavour of the fish [24].

In Zimbabwe, there have been several studies on fish parasites. These include the studies in Lake Kariba [25–29]. In these studies, *Contracaecum* larvae were present in the body cavity and intestines of siluriform and cichlid fishes, as well as the tigerfish (*Hydrocynus vittatus*) [4]. Douellou and Erlwanger [28] recovered *Clinostomum* metacercaria in nonsiluroid fishes in Lake Kariba. Magadza [30] reported the occurrence of *Clinostomum* in *Oreochromis mossambicus* and *Tilapia rendalli* in Lake Kariba. A study of *Centrocestus formosanus* in *Barbus fasciolatus* was done by Beverly-Burton [31] from Mazowe and Kadoma, while Khalil and Polling [4] recorded the occurrence of the nematodes *Contracaecum* larvae and *Procamallanus laevionchus* in *Clarius gariepinus* from Lake Kariba.

Zimbabwe has no natural lakes but has many man-made reservoirs, many of which support important capture fisheries. One of these is Mashoko dam in Masvingo Province. Man-made impoundments are becoming important for fish production [32].

The quality of fish from the Mashoko dam is of concern to fishermen and fish consumers. Fish consumers and fishers have reported the occurrence of parasitic larvae in breams (cichlids) from Mashoko dam. Due to these concerns by fishers and consumers, it was necessary to study the occurrence of yellow grub (*Clinostomum*) in Mozambique bream (*Oreochromis mossambicus)* from Mashoko dam. The objectives of the study were to determine the following:

- Presence of *Clinostomum* larvae in *O. mossambicus* from Mashoko dam
- Mean parasite intensity (MI) of infection in *O. mossambicus*
- The relationship between parasitic burden and fish sex and size (total length)
- The monthly mean condition factor of infected and uninfected *O. mossambicus.*

## 2. Materials and Methods

Mashoko dam is a man-made impoundment created in 1994 by the damming of Chenjere River [33]. The dam was constructed with the goal of providing water for irrigation, for domestic use, and for livestock [33]. It is located at geographical coordinates, Latitude 20°483′S, and Longitude 31°767′E, and has a width of 790 m, length of 1.5 km, and a wall height of 19.2 m [23]. The dam has a catchment area of 1 536 km^2^ and a volume of 1.5 × 10^6^ m^3^ at full capacity, with a surface area of approximately 70 hectares [33].

Sampling was conducted twice every month for a period of six months (June to November 2016). Fish were caught by licensed artisanal local fishermen using canoes and gill nets. The gill nets used were 30 m long with a mesh size of 76 mm. Gill nets were set overnight. The nets were set between 1700 hours and 1830 hours and were retrieved the next morning between 0600 hours and 0800 hours.

Data collected for each individual fish included Total Length (TL), Standard Length (SL), sex and wet weight (g), and infection status of each fish by *Clinostomum* larvae. Statistical computations of monthly mean parasite intensity, prevalence, and abundance were carried out using Microsoft Excel (2013**)**. Pearson's correlation analysis was used to determine the relationship between parasite burden and fish length. The relationship between parasite burden and sex was determined using Student's *t*-test in SPSS version 20. All the statistical analyses were carried out at 95% confidence interval. The monthly condition factors (cf) of infected and uninfected fish were determined using the equation

(1) $$\begin{array}{c} \text{cf}\;\;=\frac{\left(W\;\times \;100\right)}{{\text{SL}}^{3}}, \end{array}$$

where *W* = weight in grams (wet/ungutted weight) and SL = standard length in centimetres [34].

## 3. Results and Discussion

A total of 180 individuals of *Oreochromis mossambicus* (101 females and 79 males) were caught during the study. All the fish were examined for the presence of *Clinostomum* parasites. One hundred and thirteen fish specimens were found infected by 284 *Clinostomum* metacercariae.

The occurrence of *Clinostomum* larvae primarily reflects the type of habitat of their intermediate hosts (snails) or final hosts (birds). Parasites are a natural component of the environment, present in all ecosystems [34]. Aquatic habitats offer ideal conditions for the maintenance and evolution of parasite life cycles [35]. Water provides a physiologically stable and buffered environment for parasites [36]. The viscosity of water facilitates dispersal and survival of parasite eggs and their fragile free-living stages [37].

Food webs in aquatic ecosystems are relatively long and intricate and this has, in many cases, enabled the development of complex parasite life cycles [38]. Fish play an important role as consumers in aquatic food chains and also offer a large surface area for encounter and colonisation which allow them to be frequently utilized as hosts by parasitic organisms [38]. In addition, fishes are highly mobile, and this may be attractive to some parasites since they create the potential for further dispersal [39]. Due to these factors the occurrence of parasites is a common phenomenon in aquatic ecosystems [38]. Parasites of Clinostomum were prevalent in *O. mossambicus* as shown in Table 1.

Conditions in water such as oxygen content and salinity, coupled with pollutants, directly or indirectly influence both the prospective host and the parasite, but in most cases, they enhance survival of the parasite [35]. Man-made open waters (dams) represent more suitable environments for completion of digenean trematode life cycles than riverine habitats [40]. Dams also provide conditions such as higher water temperature and lower water velocity that encourage the presence of snails and birds [41].

Of the 180 specimens of *O. mossambicus* collected during this study, 113 (62.8%) were infected with *Clinostomum* larvae. A total of 284 parasites were recovered during the study. A mean intensity (MI) of 2.5 worms per fish and a maximum intensity of 16 worms per fish were recorded.

More worms were recovered from female fish than males (Table 2). Female fish had a higher prevalence percentage of infection than males. The study also revealed that female *O. mossambicus* had a greater parasitic load than males. Rohde [42] noted that endoparasites infest fish sexes differently. Helminths are mostly found in freshwater fishes where factors such as parasite and its biology, host and its feeding habitats, physical factors, and presence of intermediate hosts contribute to their prevalence and intensity [43, 44]. The results in this study were different from the findings of earlier researchers in some freshwater fish species. For instance, more males than females of *Oreochromis niloticus* and *Tilapia zilli* from the Asa dam, north central Nigeria, were infected with *Clinostomum tilapia* larvae [45].

The results of the *t*-test showed that mean intensity of infection was not significantly different between the sexes (*n*=180; *t*=0.521; *p* < 0.05). In a study of *Neutra clinostomum intermedial* in *O. mossambicus* in the Middle Letaba dam in South Africa, there was no marked difference in the infestation rates between the male and female fish [18].

The location of Mashoko dam renders it susceptible to pollution by inorganic fertilisers from the communal areas and sewage effluent from the police camp, business centre, and teachers' cottages. This pollution could have favoured the prevalence of the parasites. Fish in polluted waters tend to harbour more endoparasites than those in less or unpolluted environments [46, 47]. This is likely due to increased physiological stress in the fish due to the constant exposure to poorer quality water [47].

The relationship between *Clinostomum* burden and total length of *O. mossambicus* was significant (*r*^2^=0.76; *p* < 0.05). Previous studies on the relationship between parasite burden and size of fish (total length and weight) have reported contrasting results. In some cases, there was a positive correlation [48], while in other studies, there was no correlation [49].

Uninfected fish were in poorer condition than infected fish in July and October only (Table 3). In this study, the breeding stage of fish was not recorded. Therefore, it was not possible to determine the condition of the fish during the breeding season as condition is lower soon after breeding. The low condition factor in October may have been related to breeding activities. Apart from October, the monthly condition factor did not show any definite trend in the other months. Since sampling was conducted for only 6 months, it was not possible to come up with definite conclusions regarding the annual cycle. Some studies have reported a higher prevalence in the dry season than the wet season, and this was attributed to habitat contraction [19]. Other studies of the occurrence of *Clinostomum* metacercariae in Mozambique bream *O. mossambicus* have reported no significant seasonal difference in infestation [18].

The lowest mean condition factor for both infected and uninfected fish occurred during the month of October. In a study in Malilangwe reservoir, south-eastern lowveld in Zimbabwe, mean condition factor (K) values were greater than one (1.34–9.29) for *O. mossambicus*, *O. placidus*, and *O. macrochir*, *C. gariepinus*, and *L. altivelis*, while it was less than one for *H. vittatus* (0.82–3.09) [50]. Condition factors greater than 1 suggest that fish are in a healthy condition [50]. In the current study, the mean condition factor (mean cf.) values obtained for both infected (1.9–3.3) and uninfected (1.8–3.4) *O. mossambicus* were greater than one. The condition factors observed in this study were similar to those obtained in Malilangwe reservoir in Zimbabwe.

The results indicate that, although *O. mossambicus* was infected with *Clinostomum* parasites, it still had good condition. The infected fish were in good condition in Mashoko dam. Barson [51] observed that, even though heavy infestation in fish did not affect the condition of the host, it may render the fish unsightly and unsuitable for human consumption especially if the larvae encysted in the muscle tissues.

Fish also lose condition as they mobilise energy reserves for reproduction. The monthly condition of most fish species declines during summer [52]. He concluded that there are seasonal differences in condition of fish. A seasonal variation in fish condition was not clearly observed in the current study because the fish were sampled for only 6 months of the year. The condition factor of fish usually goes through an annual cyclical pattern [50].

In this study, there was a positive correlation between parasite burden and size of fish (Table 4). A similar correlation was observed by Price and Clancy [48]. Several studies have also reported increased parasite prevalence and intensity with increased fish size [11, 18, 19].

In a study of *Clinostomum* metacercaria in *Schilbe intermedius*, the prevalence of parasites increased gradually with increased fish size, while the mean intensity was highest in the smallest length group and lowest in the largest length group [53]. In other studies, there was no correlation [49].

## 4. Conclusion and Recommendations

The results show that *Clinostomum* metacercariae are important helminth parasites of *O. mossambicus* in Mashoko dam with 62.8% of sampled fish having been infected [54]. More parasites and greater mean parasite intensity occurred in female than in male fish. The difference in mean intensity of infection between male and female fish was not statistically significant. There was a positive correlation between parasite burden and fish size (total length). The monthly mean condition of *O. mossambicus* was not significantly different between infected and noninfected fish.

It is recommended that further studies of the occurrence of parasites be carried out for at least two full years so as to capture any possible cyclical and annual patterns. The water quality of Mashoko dam should also be studied to determine both the physicochemical and bacteriological characteristics since the dam receives run-off from farms as well as domestic effluent from the surrounding community. Further studies should also compare parasite occurrence in Mashoko dam (lentic environment) and the Chenjere River (lotic environment).

## Figures and Tables

**Table 1:** The prevalence, mean intensity, and abundance of *Clinostomum* in *O. mossambicus* (*n*=284).

Parasites	No. of infected fish	Prevalence (%)	Mean intensity (SD)
*Clinostomum* species	113	62.8	2.5 ± 0.95

**Table 2:** Prevalence and mean intensity of *Clinostomum* in female and male *O. mossambicus*.

Sex	Number of hosts examined	Number of hosts infected	Number of parasites recovered	Prevalence (%)	Mean intensity
Female	101	67	183	66.3	2.7
Male	79	46	101	58.2	2.2

**Table 3:** Monthly mean condition factors for infected and uninfected *O. mossambicus* from June to November 2016.

Month	Fish status	Mean condition factor	Standard dev
June	Infected	3.1	0.8
June	Uninfected	3.4	1.1
July	Infected	3.3	1
July	Uninfected	3	0.6
August	Infected	3.1	0.4
August	Uninfected	3.1	0.4
September	Infected	3.1	0.4
September	Uninfected	3.1	0.8
October	Infected	1.9	0.3
October	Uninfected	1.8	0.5
November	Infected	3.1	0.2
November	Uninfected	3.1	0.2

**Table 4:** Correlation between *Clinostomum* burden and total length of *O. mossambicus* (*r*^2^=0.76).

Parasite species	Fish species	*r* value
*Clinostomum*	*O. mossambicus*	0.87

## Data Availability

The data used to support the findings of this study are available from the corresponding author upon request.
